# Full-Duplex Multi-Hop Wireless Networks Optimization with Successive Interference Cancellation

**DOI:** 10.3390/s18124301

**Published:** 2018-12-06

**Authors:** Lei Shi, Zhehao Li, Xiang Bi, Lulu Liao, Juan Xu

**Affiliations:** School of Computer Science and Information Engineering, Hefei University of Technology, Hefei 230601, China; shilei@hfut.edu.cn (L.S.); lzh199501@163.com (Z.L.); llulu123@163.com (L.L.); xujuan@hfut.edu.cn (J.X.)

**Keywords:** in-band full-duplex, successive interference cancellation, multi-hop wireless network, interference management

## Abstract

In wireless network communication, in-band full-duplex technique is a useful and important technique that can enlarge the whole throughput of the wireless networks. However, its use needs harsh environment. The successive interference cancellation can make several transmitters’ data be received simultaneously by the receiver, and can make the in-band full-duplex technique be used easily in reality. In this paper, we try to propose an optimal algorithm for increasing the throughput of full-duplex multi-hop wireless networks with successive interference cancellation, which we call the full-duplex successive interference cancellation (FD-SIC) wireless networks. We first describe the mathematical model for the FD-SIC wireless networks and show it is NP-hard in general. Then, we propose a heuristic algorithm, namely the use-up-link-capacity iterative (UULC-iterative) algorithm, for each node’s routing and transmitting scheme. Simulation results show that the proposed algorithm for FD-SIC wireless networks can achieve better throughput compared with SIC-only networks and the interference avoidance networks.

## 1. Introduction

In traditional study, wireless network communication is considered as working in half-duplex (HD) model or out-of-band full-duplex model [1]. Researchers believe the wireless node cannot transmit and receive simultaneously because of the existence of strong self-interference [2]. Based on this assumption, the traditional research work on wireless network communication is often modeled into HD model [3,4,5]. Fortunately, recent advances in self-interference cancellation have made the in-band full-duplex (FD) model based wireless communication become reality. The power associated with residual self-interference can be effectively canceled for feasible in-band full-duplex transmission with combinations of various advanced analog, passive, and digital self-interference cancellation schemes [6]. In-band full-duplex operation has emerged as an attractive solution for increasing the throughput of wireless communication networks [7]. The first paper about FD wireless communication can be found in [8], where the authors propose a novel technique named antenna cancellation to fulfill self-interference cancellation. After that, many researchers have done their work on this field [9,10]. For example, in [11], according to antenna theory, the authors proposed an improved model of the antenna cancellation scheme for decoding self interference. In [12], the authors used digital cancellation to achieve Self-Interference Cancellation. In [13], simulation results show the self-interference cancellation can remove most of the self interference such that the residual interference may be regarded as mere additional noise. In [14,15], the author comprehensively analyzed the advantages and disadvantages of FD technology, and pointed out a variety of new directions and open problems associated with FD technology.

**Figure 1:**
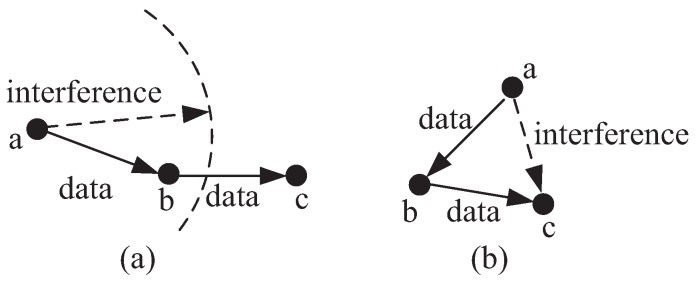
An example of the interference in the FD model. (**a**) node *c* is not in the interference range of node *a* (**b**) node *c* is in the interference range of node *a*.

Wireless communication environment is a complex environment and the use of FD technique may make the problem more complex. For example, in [16], the authors indicated that even two links, a→b and c→d, which can be active simultaneously in a HD fashion, may not be active in a FD fashion, because the link b→a may interference the link c→d. Then, the authors supposed that each link can operate in either FD or HD model, and proposed an algorithm based on a Bayesian game for each link’s operated model selection. In our paper, we consider an environment in which nodes will transmit their data to base stations by multi-hop. Under this circumstance, a node may receive from one node and transmit to another node simultaneously by the FD fashion. An example is shown in Figure 1. In Figure 1a, node *a* transmits to node *b* while node *b* transmits to node *c* simultaneously. Since node *c* is not in the interference range of node *a*, both transmissions can be successful. In Figure 1b, since node *c* is in the interference range of node *a*, the FD fashion cannot be fulfilled directly here.

We can certainly solve this problem by proposing an algorithm, such as that in [16]. However, if the network topology is in a dense environment with many wireless nodes, then it might be found that, in most cases, node *a*’s transmission to node *b* will interference with node *b*’s transmission to node *c*. In other words, in most cases, FD fashion cannot be fulfilled under this environment. Fortunately, nowadays, new techniques for interference management (IM) [17] give us new opportunities for solving this problem. IM is not a single technique; many techniques can fulfill the interference management, such as interference alignment (IA) [18], interference coordination [19], etc. The key idea of IM is trying to let the receivers receive several transmissions successfully by some signal processing methods. For example, if a receiver has the ability of successive interference cancellation (SIC) [20], then when it receives a combined data from several transmitters, it will first try to decode the strongest signal in the combined data. The requirement for decoding the strongest signal successfully is that the signal-to-interference noise ratio (SINR) is larger than a threshold. After receiving the strongest signal, the receiver will remove it from the initial combined data and then get new combined data. The processing can be repeated until all signals are decoded or one combined data cannot be decoded.

The SIC technique is a valuable technique in IM and has aroused many researchers’ interests. For example, in [21], the authors proposed a cross-layer optimization framework for multi-hop wireless networks with SIC, and showed that, compared with the scheme without SIC, the network throughput can be increased about 47%. In [22], the authors proposed a heuristic algorithm for routing in multi-hop wireless networks, and getting a bandwidth-aware high-throughput protocol with SIC. Simulation results show the proposed algorithm has about 29–62% higher throughput than the compared algorithm. In [23], the authors used the SIC technique in a special wireless network: the device-to-device-enabled (D2D-enabled) cellular network. They presented an analytical framework for studying the performance of SIC in this special network, and derived some general expressions for the successful transmission probabilities in SIC. In [24], the authors used the SIC technique in multiple-input multiple-output (MIMO) relay systems to eliminate loop interference. Simulation results show that the proposed method significantly improves the performance of FD-MIMO relay systems. We proposed an algorithm for multi-hop wireless network in [25] and an optimal base station placement algorithm with SIC in [26], and tried to use SIC technique in a special wireless network environment: the mine locomotive wireless network [27]. We found the SIC technique can make the FD fashioned wireless network more powerful. Thus, in this paper, we design an algorithm for full-duplex multi-hop wireless networks with successive interference cancellation. In the following, we call this network scheme as the FD-SIC multi-hop networks.

The following is the structure of the paper. In Section 2, we give the mathematical model for FD-SIC multi-hop wireless networks. We first give the physical layer and link layer model, then give the network layer and finally deduce the problem formulation. The problem formulation is a NILP problem. In Section 3, we propose a heuristic algorithm for solving the problem deduced in Section 2. We first give the main idea of the proposed algorithm, and then discuss some key steps in the algorithm carefully. Section 4 shows the simulation results for the proposed algorithm. We first give results for a special network with 10 nodes and 2 base stations, and then give more results for more networks. We compare results with the SIC only scheme and with interference avoidance scheme, and show that the FD-SIC scheme can achieve better throughput. In Section 5, we conclude the whole paper.

## 2. The Mathematical Model for FD-SIC Multi-Hop Wireless Networks

**Figure 2:**
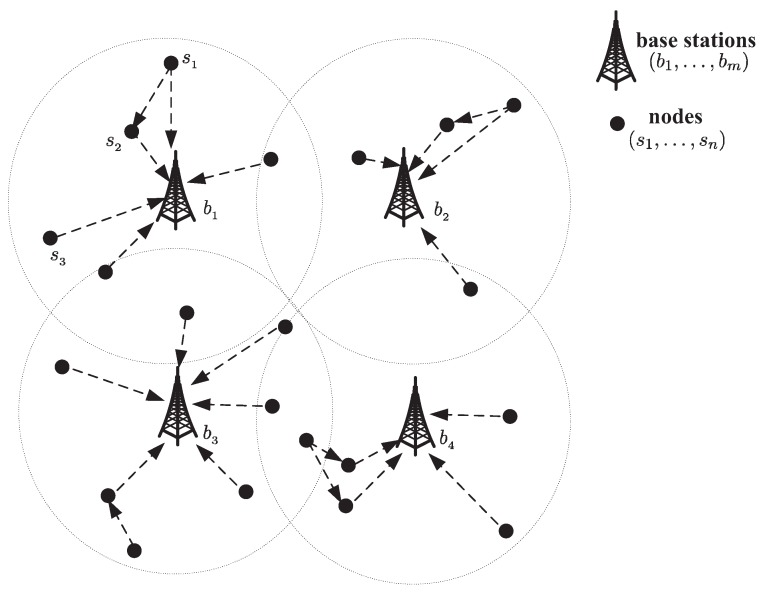
Topological structure of a multi-hop random network.

We first describe the throughput maximization problem for FD-SIC multi-hop wireless networks and give the mathematical model. Consider a FD-SIC multi-hop wireless network in a two-dimensional area with *n* nodes $s_{1},s_{2},\cdots ,s_{n}$ and *m* base stations $b_{1},b_{2},\cdots ,b_{m}$ (see Figure 2). Nodes transmit data to the base stations via single- or multi-hop transmissions, and during the whole scheduling time, each node communicates with only one base station. Suppose that all nodes have the same transmission power *P* and bandwidth *W*. Denote *N* as the set of *n* nodes and *M* as the set of *m* base stations.

### 2.1. Physical Layer and Link Layer Model

In the physical layer, we use the FD-SIC technique. In the link layer, we consider a time schedule scheme. Suppose the whole schedule time *T* is divided into *h* time slots equally, and denote $t_{k}\left(k=1,\cdots ,h\right)$ as these time slots. Define a binary scheduling variable $x_{s_{i}\to s_{j}}^{k}$ for link $s_{i}\to s_{j}$ in time slot $t_{k}$, and a binary scheduling variable $x_{s_{i}\to b_{j}}^{k}$ for link $s_{i}\to b_{j}$ in time slot $t_{k}$. We have,

$$x_{s_{i}\to s_{j}}^{k}=\left\{\begin{array}{cc} 1: & \mathrm{if}\;\mathrm{node}\;s_{i}\;\mathrm{transmits}\;\mathrm{data}\;\mathrm{to}\;\mathrm{node}\;s_{j}\;\mathrm{in}\;\mathrm{time}\;\mathrm{slot}\;t_{k};\\ 0: & \mathrm{otherwise}. \end{array}\right.$$

$$x_{s_{i}\to b_{j}}^{k}=\left\{\begin{array}{cc} 1: & \mathrm{if}\;\mathrm{node}\;s_{i}\;\mathrm{transmits}\;\mathrm{data}\;\mathrm{to}\;\mathrm{base}\;\mathrm{station}\;b_{j}\;\mathrm{in}\;\mathrm{time}\;\mathrm{slot}\;t_{k};\\ 0: & \mathrm{otherwise}. \end{array}\right.$$

In a time slot $t_{k}$, a node $s_{i}$ can transmit to at most one node (or one base station), i.e.,

(1) $$\begin{array}{cc} \sum_{s_{j}\in T_{s_{i}}}x_{s_{i}\to s_{j}}^{k}+\sum_{b_{l}\in T_{s_{i}}}x_{s_{i}\to b_{l}}^{k}\leq 1 & (s_{i}\in N,1\leq k\leq h)\;. \end{array}$$

If we use HD model, then node $s_{i}$ cannot transmit or receive simultaneously:

(2) $$\begin{array}{cc} \frac{\sum_{s_{m}\in T_{s_{i}}}x_{s_{m}\to s_{i}}^{k}}{|T_{s_{i}}|}+\sum_{s_{j}\in T_{s_{i}}}x_{s_{i}\to s_{j}}^{k}\leq 1 & (s_{i}\in N,1\leq k\leq h)\;. \end{array}$$

In FD model, the equation will become,

(3) $$\begin{array}{cc} \frac{\sum_{s_{m}\in T_{s_{i}}}x_{s_{m}\to s_{i}}^{k}}{|T_{s_{i}}|}+\sum_{s_{j}\in T_{s_{i}}}x_{s_{i}\to s_{j}}^{k}\leq 2 & (s_{i}\in N,1\leq k\leq h)\;. \end{array}$$

where $T_{s_{i}}$ is the set of all neighbors in the transmission range $R_{T}$ of node $s_{i}$ in Equations (1)–(3).

When using SIC, the receiver can receive multiple signals simultaneously and decode them sequentially. Based on the SIC decoding process, we can find that, when node $s_{j}$ (or base station $b_{j}$) tries to decode the signal received from node $s_{i}$ in a time slot $t_{k}$, all stronger signals have already been decoded and removed.Thus, we will have an improved SINR, which, to ensure node $s_{j}$ (or base station $b_{j}$) can decode signal from node $s_{i}$, should be no less than β. Denote the channel model $g=d^{-3}$, where *d* is the distance between two nodes. Denote $N_{0}$ as the noise power and $N_{SI}$ as the node’s residual self-interference after using the FD technique. Note that all nodes use the same transmission power *P* and thus the residual self-interference $N_{SI}$ is a constant. Then, we have,

(4) $$\begin{array}{c} SINR_{s_{i}\to s_{j}}^{k}=\frac{g_{s_{i}\to s_{j}}P}{\sum_{s_{l}\neq s_{i}}^{g_{s_{l}\to s_{j}}\leq g_{s_{i}\to s_{j}}}\left(g_{s_{l}\to s_{j}}P\sum_{s_{m}\in T_{l}}x_{s_{l}\to s_{m}}^{k}\right)+N_{0}+N_{SI}}\geq \beta \;\;\left(x_{s_{i}\to s_{j}}=1\right)\;, \end{array}$$

(5) $$\begin{array}{c} SINR_{s_{i}\to b_{j}}^{k}=\frac{g_{s_{i}\to b_{j}}P}{\sum_{s_{l}\neq s_{i}}^{g_{s_{l}\to b_{j}}\leq g_{s_{i}\to b_{j}}}\left(g_{s_{l}\to b_{j}}P\sum_{s_{m}\in T_{l}}x_{s_{l}\to s_{m}}^{k}\right)+N_{0}+N_{SI}}\geq \beta \;\;\left(x_{s_{i}\to b_{j}}=1\right)\;, \end{array}$$

where $\sum_{s_{m}\in T_{l}}x_{s_{l}\to s_{m}}^{k}=1$ if $s_{l}$ is transmitting data to some node in time slot $t_{k}$ and $\sum_{s_{m}\in T_{l}}x_{s_{l}\to s_{m}}^{k}=0$ if $s_{l}$ is not transmitting in time slot $t_{k}$. Here, we suppose $x_{s_{i}\to s_{j}}=1$ (or $x_{s_{i}\to b_{j}}=1$), because if $x_{s_{i}\to s_{j}}=0$ (or $x_{s_{i}\to b_{j}}=0$), it means $s_{i}$ does not transmit to $s_{j}$ (or $b_{j}$) at that time and we do not need to consider this situation.

### 2.2. Network Layer Model and Problem Formulation

Now, we give the network layer model. Suppose each node $s_{i}$ has a minimum data rate requirement $r_{i}$ to one base station. We want to maximize a common scaling factor *K* [28] such that each $s_{i}$ can transmit its data to the base station with a data rate $Kr_{i}$. Denote $r_{s_{i}\to s_{j}}$, $s_{i}\in N,s_{j}\in T_{s_{i}}$, as the average data rate from node $s_{i}$ to node $s_{j}$. Denote $r_{s_{i}\to b_{j}}$, $s_{i}\in N,s_{i}\in T_{b_{j}}$, as the average data rate from node $s_{i}$ to base station $b_{j}$. Then, we have the following relationship for flow rates at a node $s_{i}$,

(6) $$\begin{array}{cc} \sum_{s_{l}\in T_{s_{i}}}r_{s_{l}\to s_{i}}+Kr_{i}=\sum_{s_{j}\in T_{s_{i}}}r_{s_{i}\to s_{j}}+\sum_{s_{i}\in T_{b_{j}}}r_{s_{i}\to b_{j}} & (s_{i}\in N)\;. \end{array}$$

Since, for each link, the flow rate cannot exceed the achievable average link rate, we have,

(7) $$\begin{array}{cc} r_{s_{i}\to s_{j}}\leq \frac{1}{h}\sum_{k=1}^{h}\left(C\cdot x_{s_{i}\to s_{j}}^{k}\right) & (s_{i}\in N,s_{j}\in T_{s_{i}})\;, \end{array}$$

(8) $$\begin{array}{cc} r_{s_{i}\to b_{j}}\leq \frac{1}{h}\sum_{k=1}^{h}\left(C\cdot x_{s_{i}\to b_{j}}^{k}\right) & (s_{i}\in N,s_{i}\in T_{b_{j}})\;, \end{array}$$

where *C* is the data rate by a successful transmission, which can be calculated by $Wlog_{2}\left(1+\beta \right)$. Here, we consider a fixed modulation and coding scheme [29], which achieves a fixed transmission rate *C* if successful. The adaptive modulation and coding scheme, which achieves a transmission rate as a function of SINR, is also considered by many other papers. However, it requires accurate estimation on channel condition. If there are errors on channel condition estimation and a high transmission rate is used, such a transmission may not be decoded due to small SINR and thus the achieved rate is 0. Due to this risk, many systems use a fixed modulation and coding scheme and we also consider this scheme in the paper. In this paper, we consider the fixed modulation and coding scheme.

Based on these discussions, the problem can be formulated as follows.

(9) $$\begin{array}{c} \begin{array}{ccc} \max & K & \\ s.t. & \sum_{s_{j}\in T_{s_{i}}}x_{s_{i}\to s_{j}}^{k}+\sum_{b_{l}\in T_{s_{i}}}x_{s_{i}\to b_{l}}^{k}\leq 1 & \left(s_{i}\in N,1\leq k\leq h\right)\\ & \frac{\sum_{s_{m}\in T_{s_{i}}}x_{s_{m}\to s_{i}}^{k}}{|T_{s_{i}}|}+\sum_{s_{j}\in T_{s_{i}}}x_{s_{i}\to s_{j}}^{k}\leq 2 & \left(s_{i}\in N,1\leq k\leq h\right)\\ & SINR_{s_{i}\to s_{j}}^{k}=\frac{g_{s_{i}\to s_{j}}P}{\sum_{s_{l}\neq s_{i}}^{g_{s_{l}\to s_{j}}\leq g_{s_{i}\to s_{j}}}\left(g_{s_{l}\to s_{j}}P\sum_{s_{m}\in T_{l}}x_{s_{l}\to s_{m}}^{k}\right)+N_{0}+N_{SI}}\geq \beta & \left(x_{s_{i}\to s_{j}}=1\right)\\ & SINR_{s_{i}\to b_{j}}^{k}=\frac{g_{s_{i}\to b_{j}}P}{\sum_{s_{l}\neq s_{i}}^{g_{s_{l}\to b_{j}}\leq g_{s_{i}\to b_{j}}}\left(g_{s_{l}\to b_{j}}P\sum_{s_{m}\in T_{l}}x_{s_{l}\to s_{m}}^{k}\right)+N_{0}+N_{SI}}\geq \beta & \left(x_{s_{i}\to b_{j}}=1\right)\\ & \sum_{s_{l}\in T_{s_{i}}}r_{s_{l}\to s_{i}}+Kr_{i}=\sum_{s_{j}\in T_{s_{i}}}r_{s_{i}\to s_{j}}+\sum_{s_{i}\in T_{b_{j}}}r_{s_{i}\to b_{j}} & \left(s_{i}\in N\right)\\ & r_{s_{i}\to s_{j}}\leq \frac{1}{h}\sum_{k=1}^{h}\left(C\cdot x_{s_{i}\to s_{j}}^{k}\right) & \left(s_{i}\in N,s_{j}\in T_{s_{i}}\right)\\ & r_{s_{i}\to b_{j}}\leq \frac{1}{h}\sum_{k=1}^{h}\left(C\cdot x_{s_{i}\to b_{j}}^{k}\right) & \left(s_{i}\in N,s_{i}\in T_{b_{j}}\right)\\ & x_{s_{i}\to s_{j}}^{k},x_{s_{i}\to b_{j}}^{k}\;\in \;\left\{0,1\right\},r_{s_{i}\to s_{j}},r_{s_{i}\to b_{j}},K\;\geq \;0 & (s_{i}\;\in \;N,s_{j}\;\in \;T_{s_{i}},1\;\leq \;k\;\leq \;h). \end{array} \end{array}$$

In Equation (9), *K*, $x_{s_{i}\to s_{j}}^{k}$, $x_{s_{i}\to b_{j}}^{k}$, and $r_{s_{i}\to b_{j}}$ are variables. This problem model is a mixed integer linear programming (MILP) problem, which is NP-hard in general [30] and cannot be solved directly.

## 3. Optimization for FD-SIC Multi-Hop Wireless Networks

In Section 2, we give the problem formulation and show it is a MILP problem, which is NP-hard in general. In other words, it is very challenging to find an optimal solution for FD-SIC multi-hop wireless networks. In this section, we propose a heuristic algorithm for solving the problem. Notice that the main step of the heuristic algorithm is based on the iterative idea, and, in each iterative step, we always want to try use up all link capacity, so we call our algorithm as the use-up-link-capacity iterative (UULC-iterative) algorithm.

### 3.1. Main Idea

It is easy to find that in Equation (9), binary scheduling variables $x_{s_{i}\to s_{j}}^{k}$ and $x_{s_{i}\to b_{j}}^{k}$ can make the problem non-convex and thus NP-hard in general. If we can find a way to determine these values, then the remaining problem is only with continuous variables $r_{s_{i}\to s_{j}}$,$r_{s_{i}\to b_{j}}$ and *K*, i.e.,

(10) $$\begin{array}{c} \begin{array}{ccc} \max & K & \\ s.t. & \sum_{s_{l}\in T_{s_{i}}}r_{s_{l}\to s_{i}}+Kr_{i}=\sum_{s_{j}\in T_{s_{i}}}r_{s_{i}\to s_{j}}+\sum_{s_{i}\in T_{b_{j}}}r_{s_{i}\to b_{j}} & \left(s_{i}\in N\right)\\ & r_{s_{i}\to s_{j}}\leq \frac{1}{h}\sum_{k=1}^{h}\left(C\cdot x_{s_{i}\to s_{j}}^{k}\right) & \left(s_{i}\in N,s_{j}\in T_{s_{i}}\right)\\ & r_{s_{i}\to b_{j}}\leq \frac{1}{h}\sum_{k=1}^{h}\left(C\cdot x_{s_{i}\to b_{j}}^{k}\right) & \left(s_{i}\in N,s_{i}\in T_{b_{j}}\right)\\ & r_{s_{i}\to s_{j}},r_{s_{i}\to b_{j}},K\geq 0 & (s_{i}\in N,s_{j}\in T_{s_{i}},1\leq k\leq h)\;. \end{array} \end{array}$$

This formulation is a linear programming (LP) problem, and can be solved in polynomial-time. To do that, we need first establish a way to determine the value of each $x_{s_{i}\to s_{j}}^{k}$ and $x_{s_{i}\to b_{j}}^{k}$. Then, based on this idea, we can propose a heuristic algorithm based on iterative framework for solving the whole problem. The following four steps are the main idea for determining these values.

I Divide the whole area into several regions. Each region includes one base station and many sensor nodes. Each sensor node will only communicate with the base station in the same region, and base stations can communicate with each other.
II. For each node, establish a first routing path to the base station in its region and assign time slots for each link on this path, such that Equations (1), (3), (4) and (5) hold.
III. Under current $x_{s_{i}\to s_{j}}^{k}$ and $x_{s_{i}\to b_{j}}^{k}$ values, calculate *K*, all $r_{s_{i}\to s_{j}}$ and $r_{s_{i}\to b_{j}}$ values by Equation (10).
IV. Try to improve the current scheduling solution ($x_{s_{i}\to s_{j}}^{k}$ and $x_{s_{i}\to b_{j}}^{k}$ values), thus *K* may be increased. If we can find a way to increase *K*, then go back to Step III, else our algorithm terminates.

There are three challenges in the above algorithm: (1) how to select a base station for each node; (2) how to establish an initial path for each node; and (3) how to improve the current scheduling solution. In the following, we give the detail steps for each challenge in our algorithm.

### 3.2. Base Station Selection

**Figure 3:**
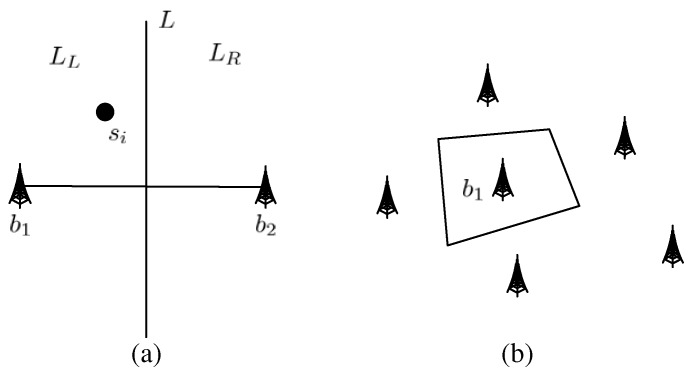
The division by the Varonoi graphic algorithm (**a**) $b_{1}$ and $b_{2}$ are two base stations in the network topology (**b**) the Varonoi polygon of $b_{1}$.

Step 1 is to select a base station for each node. To do this, we can divide the whole network topology into several regions. Each region has one base station, and nodes in this region will send their data to this base station. We use the Voronoi graphic algorithm [31] to do this division (see Figure 3), and have the following definitions.

**Definition** **1.** *Suppose $b_{1}$ and $b_{2}$ are two base stations in the network topology (see Figure 3a). L is the perpendicular bisector of the line $\bar{b_{1}b_{2}}$, and divides the whole network topology into two half-planes $L_{L}$ and $L_{R}$. Define $H(b_{1},b_{2})$ as the half-plane $L_{L}$, and $H(b_{2},b_{1})$ as the half-plane $L_{R}$.*

Obviously, to a node $s_{i}$, if $s_{i}$ is in $H(b_{1},b_{2})$, then the distance from $s_{i}$ to $b_{1}$ and $b_{2}$ should meet $d_{s_{i}\to b_{1}}<d_{s_{i}\to b_{2}}$.

**Definition** **2.** *Define $V\left(b_{i}\right)=\underset{i\neq j}{⋂}H\left(b_{i},b_{j}\right)$, where $V(b_{i})$ is an convex polygon area with no more than (n−1) edges. We call $V(b_{i})$ as the Varonoi polygon of $b_{i}$.*

For example (see Figure 3b), the Varonoi polygon of $b_{1}$ is a quadrangle, and n=6. After dividing the network topology by the Varonoi graphic algorithm, we get some regions. When a node $s_{i}\in V\left(b_{j}\right)$, it should send its data to $b_{j}$.

### 3.3. Establish the First Routing Paths

**Figure 4:**
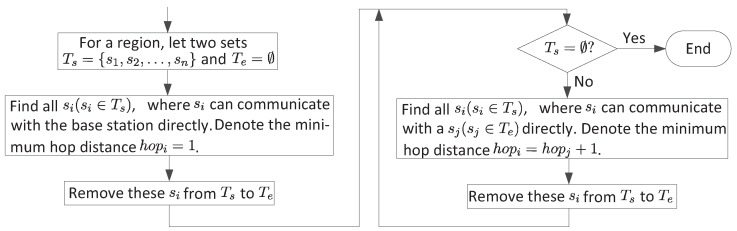
Identify the minimum hop distance for all nodes.

After dividing the network topology into several regions, we establish the first routing paths for all nodes in each region, and calculate the maximum *K* value on the current condition (Steps 2 and 3). Before that, we first identify the minimum hop distance to the base station for each node. The minimum hop distance is a usual way to calculate distances between nodes in networks. In Figure 4, we give the flow-process diagram on how to identify the minimum hop distance for all nodes.

**Figure 5:**
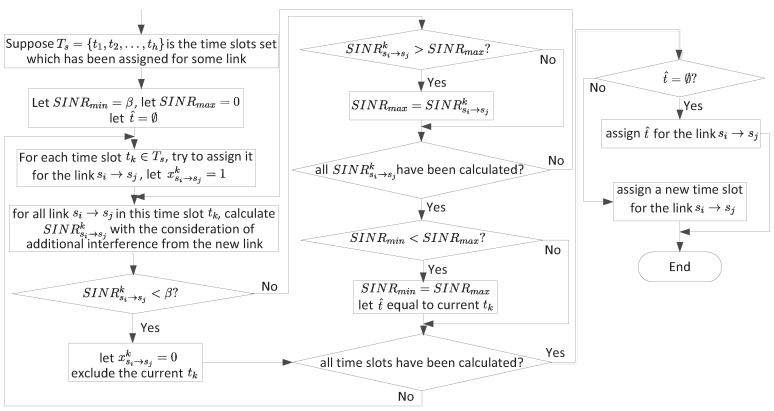
Time slot assignment for a new link $s_{i}\to s_{j}$.

We can use the minimum hop distance for establishing the first routing paths. Suppose the minimum hop distance of node $s_{i}$ is *d*, then we choose the neighboring node of $s_{i}$ with the minimum hop distance, e.g. $s_{j}$, as the next hop node. Then, a link $s_{i}\to s_{j}$ is created. In this way, we can build the first routing paths for all nodes. Then, we assign time slots for all links. The main points for assigning time slots are as follows, and the detail steps can be found in Figure 5.

- When a link $s_{i}\to s_{j}$ is created, we first try to assign a time slot already used by some other link. If this time slot cannot be used (i.e., the assignment makes some existing links or the new link with SINR<β), then we should consider another existing time slot. If no existing time slots can be used, we assign a new time slot. Otherwise, an available time slot is found.
- If we find multiple time slots for a new link, we use the minimum improved SINR (see Equations (4) and (5)) among all links in a time slot as a metric and choose the time slot that can maximize the minimum improved SINR as the most appropriate time slot.

Once the first routing paths for each node are established and all time slots for links are assigned, we can calculate the maximum *K*, $r_{s_{i}\to s_{j}}$ and $r_{s_{i}\to b_{j}}$ values by Equation (10).

### 3.4. Improve the Current Scheduling Solution

The last step is to improve the current scheduling solution. To do that, we need to identify bottleneck links and bottleneck nodes. We first give some definitions.

**Definition** **3.** *To a link $s_{i}\to s_{j}$ (or $s_{i}\to b_{j}$), define $Z_{s_{i}\to s_{j}}=\frac{1}{h}\sum_{k=1}^{h}\left(C\cdot x_{s_{i}\to s_{j}}^{k}\right)-r_{s_{i}\to s_{j}}$ (or $Z_{s_{i}\to b_{j}}=\frac{1}{h}\sum_{k=1}^{h}\left(C\cdot x_{s_{i}\to b_{j}}^{k}\right)-r_{s_{i}\to b_{j}}$) as the residual capacity on this link.*

The residual capacity on a link can be used to indicate the link’s using efficiency. Since $\frac{1}{h}\sum_{k=1}^{h}\left(C\cdot x_{s_{i}\to s_{j}}^{k}\right)$ is the maximum data rate for the link $s_{i}\to s_{j}$, $r_{s_{i}\to s_{j}}$ is the real average data rate for the link $s_{i}\to s_{j}$. Thus, the smaller $Z_{s_{i}\to s_{j}}$ is, the higher the using efficiency is.

**Definition** **4.** *To a link $s_{i}\to s_{j}$ (or $s_{i}\to b_{j}$), if there is no residual capacity on this link, i.e., $Z_{s_{i}\to s_{j}}=0$ (or $Z_{s_{i}\to b_{j}}=0$), then we call this link the bottleneck link.*

We can have similar definitions for the residual capacity on a routing path, for the residual capacity on a node, and for the bottleneck node.

**Definition** **5.** *To one routing path from a node $s_{i}$ to its base station $b_{j}$, each link on the path has its residual capacity, and we choose the minimum residual capacity on these links as the residual capacity on the routing path.*

**Definition** **6.** *To a node $s_{i}$, which may have one or several routing paths to its base station, we choose the maximum residual capacity on these routing paths as the residual capacity on the node $s_{i}$, and denote it as $Z_{s_{i}}$.*

**Definition** **7.** *To a node $s_{i}$, if its residual capacity is zero, i.e., $Z_{s_{i}}=0$, then we call this node a bottleneck node.*

The residual capacity on a node can be determined by the maximum flow algorithm [30]. It is obvious that, if we can calculate each node’s residual capacity and find the bottleneck node, we can further increase the throughput on this node by assigning an additional time slot to one of its existing links or adding a new out-going link. Thus, *K* value may also be increased. After we increase the bottleneck node’s throughput, the whole network solution will be changed and we will find a new bottleneck node. Then, we can try to increase the throughput of the new bottleneck node. This step can be repeated until we cannot increase the throughput of the bottleneck node, and we call this step the iterative step. Then, we can calculate the last maximum *K* for the whole network.

However, there are some problems we should solve in each iterative step: (1) when trying to increase the throughput of a bottleneck node, how to select from these two methods (using a new time slot on an existing link or using a new link with an assigned time slot); (2) if we use a new time slot, which time slot should be assigned on which existing link; and (3) if we use a new link, which new link should be added.

To answer these problems, we need to analyze possible improvement after using a new time slot or a new link. We consider it under the following two cases.

- *Possible improvement by a new time slot.* For each out-going link $s_{i}\to s_{j}$, we can revise the algorithm in Figure 5 to determine a suitable time slot. That is, instead of considering all time slots, we only consider time slots not used by link $s_{i}\to s_{j}$. Once a new time slot is found, possible improvement on link $s_{i}\to s_{j}$ is $\frac{C}{h}$ while possible improvement from node $s_{j}$ to the base station is $Z_{j}$. Thus, possible improvement is $\min\{\frac{C}{h},Z_{j}\}$. Note that, for the link $s_{i}\to b_{j}$, possible improvement is $\frac{C}{h}$.
- *Possible improvement by a new link.* For a new link, we can apply the algorithm in Figure 5 to determine a suitable time slot. Once a new time slot is found, possible improvement is again $\min\{\frac{C}{h},Z_{j}\}$ for the link $s_{i}\to s_{j}$, or $\frac{C}{h}$ for the link $s_{i}\to b_{j}$.

**Figure 6:**
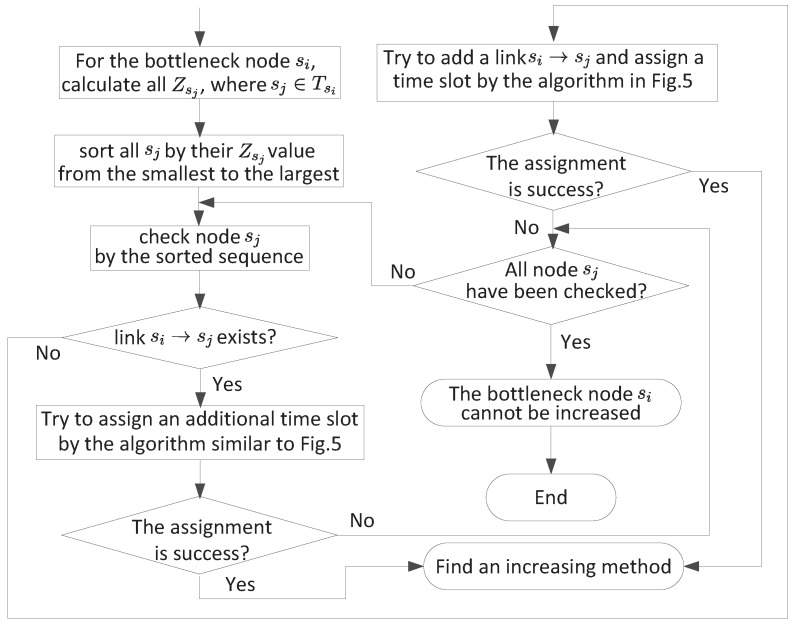
Increase bottleneck node $s_{i}$’s throughput.

Based on the above analysis, we can address the problems we have proposed. The detail steps to increase bottleneck node $s_{i}$’s throughput is given in Figure 6. Notice that, in Figure 6, we use a similar algorithm as Figure 5 to judge wether a new assignment can be successful, which means when, we want to add a new link or assign a new time slot for an exist link, all other affected nodes’ SINR should be recalculated.

### 3.5. Complexity

We first show the complexity for the base station selection step. Since we need to calculate all distances between each node and each base station to perform the Voronoi graphic algorithm, the complexity of this step is O(nm).

Then, we show that in each region the complexity in each iteration is polynomial. In the first iteration, we need to identify hop distance for all nodes, select a next hop node for each node and then assign a time slot for this link, and identify initial *K*.

- To identify hop distance for all nodes, we need to visit all neighboring nodes of each node. The complexity is $O\left(E\right)=O\left(n^{2}\right)$, where *E* is the number of all possible links.
- The complexity to select a next hop node for each node is O(n).
- The complexity to assign a time slot to the first link is O(1). To analyze the complexity to assign a time slot for the (e+1)th link, we assume that previous *e* links use $\hat{h}$ time slots. When we consider a used time slot $t_{k}$ for the (e+1)th link, we need to check up to $e_{k}+1$ SINR values, where $e_{k}$ is the number of links in this time slot. In the worst case, we may check up to $\sum_{k=1}^{\hat{h}}\left(e_{k}+1\right)=e+\hat{h}$ SINR values and then decide to assign a new time slot. The complexity is $O\left(e+\hat{h}+1\right)=O\left(e+h\right)$. Thus, the total complexity for time slot assignment is at most $O\left(1\right)+\sum_{e=2}^{E}O\left(e+h\right)=O\left(1\right)+O\left(E^{2}\right)+O\left(hE\right)=O\left(n^{4}+hn^{2}\right)$.
- The complexity of solving an LP in Equation (10) is $O(N_{v}^{3})$ where $N_{v}$ is the number of variables in Equation (10). Since $N_{v}=O\left(N^{2}\right)$, $O\left(N_{v}^{3}\right)=O\left(n^{6}\right)$.

The overall complexity in the first iteration is $O\left(n^{2}\right)+O\left(n\right)+O\left(n^{4}+hn^{2}\right)+O\left(n^{6}\right)=O\left(n^{6}+hn^{2}\right)$, which is polynomial.

In each of the subsequent iterations, we need to identify bottleneck node, calculate possible improvement of each of its neighbors, sort these neighbors, either assign a new time slot or add a new link, and update *K*.

- The complexity to identify bottleneck node is $O\left(E\right)=O\left(n^{2}\right)$.
- To calculate possible improvement for a neighboring node, we need to solve a maximum flow problem. Some algorithms (e.g., Push-relabel algorithm with FIFO vertex selection rule) for the maximum flow problem have complexity $O(n^{3})$. The total complexity for a node $s_{i}$’s $|T_{i}|$ neighbors is $|T_{i}|O\left(n^{3}\right)=O\left(n^{4}\right)$.
- Sorting $|T_{i}|$ neighbors has a complexity $O(|T_{i}|\ln|T_{i}|)=O\left(n\ln n\right)$.
- To analyze the complexity of assigning an additional time slot for an existing link, we assume that other e−1 links use $\hat{h}$ time slots. We need to check up to *e* SINR values. In the worst case, we may find that $\hat{h}$ time slots are not available and then assign a new time slot. The complexity is $O\left(\hat{h}e+1\right)=O\left(hn^{2}\right)$.
  To analyze the complexity of assigning a time slot for a new link, we assume that previous *e* links use $\hat{h}$ time slots. When we consider a used time slot, we need to check up to e+1 SINR values. In the worst case, we may find that $\hat{h}$ time slots are not available and then assign a new time slot. The complexity is $O(\hat{h}\left(e+1\right)+1=O\left(hn^{2}\right)$.
  Thus, the total complexity to assign a new time slot or add a new link is $|T_{i}|O\left(hn^{2}\right)=O\left(hn^{3}\right)$.
- The complexity of solving an LP in Equation (10) is again $O(n^{6})$.

The overall complexity in a subsequent iteration is $O\left(n^{2}\right)+O\left(n^{4}\right)+O\left(n\ln n\right)+O\left(hn^{3}\right)+O\left(n^{6}\right)=O\left(n^{6}+hn^{3}\right)$, which is polynomial.

We then analyze the number of iterations. On the one hand, in the first iteration, *n*$x_{ij}\left[k\right]$ values are set as one, while, in each subsequent iteration, one additional $x_{ij}\left[k\right]$ value is set as one. On the other hand, the total number of $x_{ij}\left[k\right]$ variables is $O\left(hE\right)=O\left(hn^{2}\right)$. Thus, the number of iterations is at most $1+O\left(hn^{2}\right)-n=O\left(hn^{2}\right)$.

Thus, the overall complexity of our algorithm is $O\left(nm\right)\left(O\left(n^{6}+hn^{2}\right)+\left(O\left(hn^{2}\right)-1\right)O\left(n^{6}+hn^{3}\right)\right)=O\left(hn^{9}m+h^{2}n^{6}m\right)$, which is polynomial. Note that the purpose of this analysis is to show the complexity is polynomial. The obtained result is a loose upper bound on complexity, while, in practice, the complexity can be much less.

## 4. Simulation Results

In this section, we give simulation results to show the performance of our algorithm. We also compare results without Full-duplex (the SIC only scheme [25]) and without SIC (the interference avoidance scheme [25]) to show the advantage of Full-duplex.

Consider multi-hop networks with 10 to 50 nodes and 2 to 5 base stations randomly deployed in a square region of 3000 m × 3000 m. Transmission power is P=1 W. Noise power and the self-interference power is $N_{0}+N_{SI}=10^{-5}$ W. The SINR threshold is β=1. Channel bandwidth is W=22 MHz. The number of time slots is equal to the number of nodes. The required minimum data rate $r(s_{i})$ is between 100 kbps and 1000 kbps. Similar parameter setting are used in [21].

We first present detailed results of a multi-hop network with 10 nodes and 2 base stations in Section 4.1. Then, we provide complete results for all network instances with different number of nodes.

### 4.1. Results for a Multi-Hop Network with 10 Nodes and 2 Base Stations

**Figure 7:**
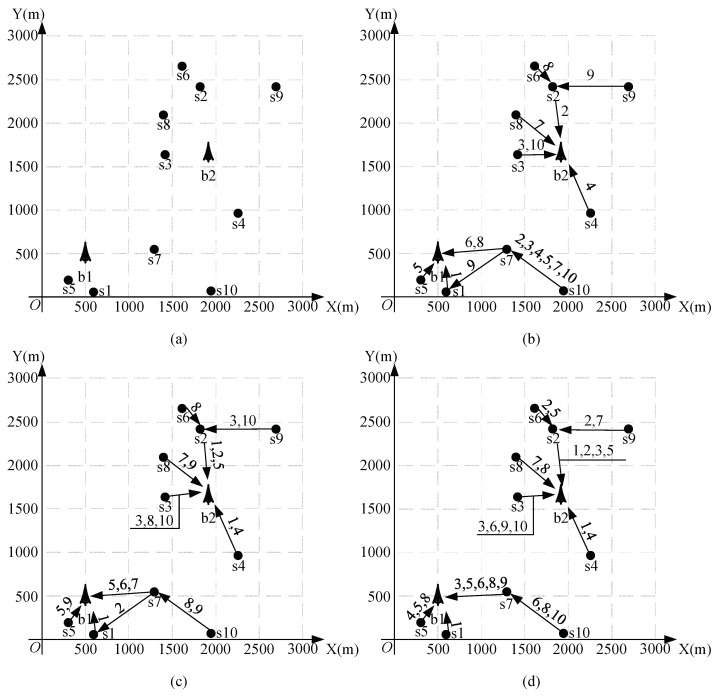
The 10-node, 2-base-station network and schemes in the three different techniques (**a**) The 10-node 2-base station wireless network. (**b**) Routing and time slot allocating scheme for interference avoidance. (**c**) Routing and time slot allocating scheme for SIC-only. (**d**) Routing and time slot allocating scheme for full-duplex.

Consider a multi-hop network shown in Figure 7a. The coordinates and the required minimum data rate $r(s_{i})$ are shown in Table 1. The two base stations’ coordinates are (500,500) and (1877,1674).

When using the Full-duplex and SIC technique, we have K=75.82; with SIC technique only, we have K=50.54; and with the interference avoidance scheme, we have K=20.21. We give the routing and the time slot allocating methods in these three different techniques in Figure 7b–d. From these we can see that, when using full-duplex, nodes will have more chances and abilities for transmitting and receiving. For example, node $s_{7}$ will transmit in five different time slots when using full-duplex (see Figure 7d), and, in time slot $t_{8}$, it will transmit to base station and receive from $s_{10}$ simultaneously. When using SIC only, $s_{7}$ will transmit in three different time slots and will not transmit and receive simultaneously. When using interference avoidance, $s_{7}$ will only transmit in two time slots.

### 4.2. Results for All Network Instances

**Figure 8:**
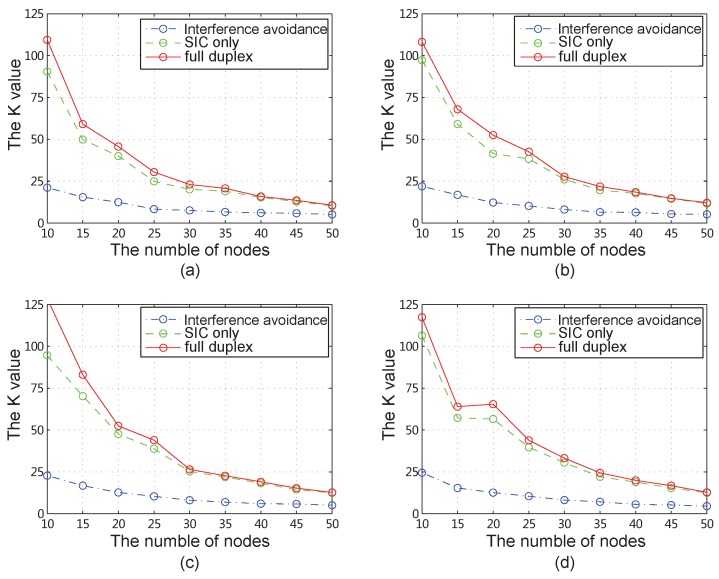
More comparisons for different number of nodes and base stations (**a**) 2 base stations. (**b**) 3 base stations. (**c**) 4 base stations. (**d**) 5 base stations.

We change the number of base stations *m* from 2 to 5, the number of nodes *n* from 10 to 50, and generate 20 different network instances randomly for each network. Then, we calculate the value *K* under the interference avoidance scheme and the SIC scheme, and show the average values for each network in Figure 8. From these simulation results, we can see that the throughput by using SIC is improved explicitly compared with using interference avoidance, and improved much more when using full duplex. Comparing with the interference avoidance scheme, the achieved throughput improvement is about 200–500% when using full duplex. The improvement is not explicit when comparing with the SIC only scheme, especially when the number of nodes is large. However, it is impactful when the number is not very big. For example, when the number of node is 20, the throughput improvement of full duplex scheme is about 16.6% comparing with the SIC only scheme.

### 4.3. Complexity from Simulation Results

**Figure 9:**
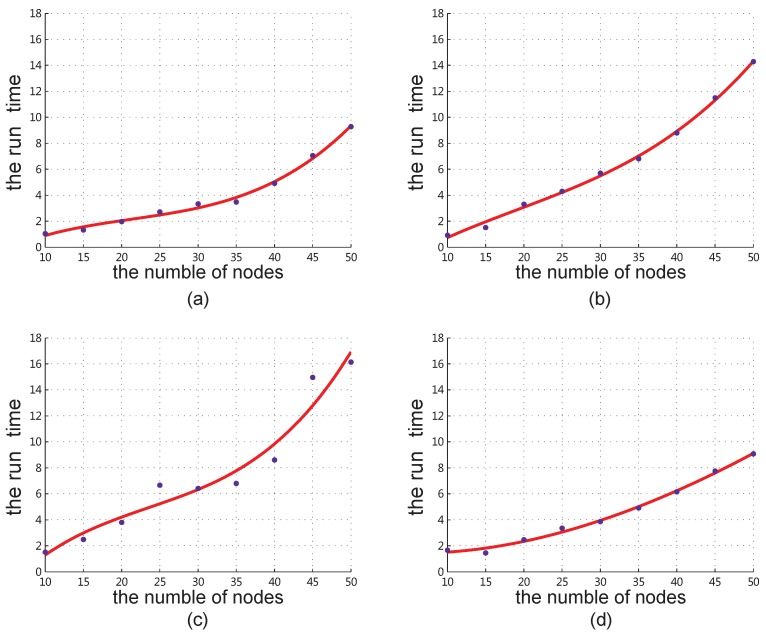
The fitting curve for the number of the nodes and the actual run-time (**a**) 2 base stations. (**b**) 3 base stations. (**c**) 4 base stations. (**d**) 5 base stations.

To get the real complexity of our algorithm, which should be less than complexity upper bound analyzed in Section 3.5, we record the run-time of each program simulation, calculate their average values, and get the fitting curve which is shown in Figure 9. The curve fitting formulas are $y=0.0002023x^{3}-0.01298x^{2}+0.3626x-1.634$ for two base stations, $y=0.0001566x^{3}-0.00901x^{2}+0.3946x-2.464$ for three base stations, $y=0.0003634x^{3}-0.02578x^{2}+0.8106x-4.601$ for four base stations and $y=-0.0001537x^{3}+0.004818x^{2}-0.05009x+1.541$ for five base stations. Compared with the original value, the fitting result has about 95% degree of similarity. The curve is a cubic polynomial curve, which means the actual time complexity of our algorithm is $\theta (n^{3})$.

## 5. Conclusions

In this paper, we consider in the wireless network environment that nodes need to transmit data to base stations by single- or multi-hop scheme. We want to use the SIC and FD techniques to increase the throughput of the whole network, and build the mathematical model which is NP-hard in general. Then, we design a heuristic algorithm, namely the UULC-iterative algorithm. Simulation results show that FD-SIC scheme by using UULC-iterative algorithm can increase the throughput obviously while comparing with the SIC only scheme and the interference avoidance scheme. 

## Figures and Tables

**Table 1:** The coordinates (in m) and the $r(s_{i})$ value (in kbps) of each node in the network.

*i*	Coordinates	$r(s_{i})$	*i*	Coordinates	$r(s_{i})$
1	(425, 462)	837.26	6	(293, 638)	302.43
2	(402, 638)	449.89	7	(617, 425)	550.01
3	(687, 711)	619.55	8	(474, 772)	578.13
4	(713, 984)	204.02	9	(995, 1017)	189.43
5	(801, 903)	112.95	10	(301, 469)	167.50
